# Biomarkers associated with the pathogenesis of Alzheimer’s disease

**DOI:** 10.3389/fncel.2023.1279046

**Published:** 2023-12-07

**Authors:** Hui Wang, Mengli Sun, Wenhui Li, Xing Liu, Mengfan Zhu, Hua Qin

**Affiliations:** ^1^College of Life Sciences, Nankai University, Tianjin, China; ^2^Research Center for Tissue Repair and Regeneration Affiliated with the Medical Innovation Research Division and 4th Medical Center, PLA General Hospital and PLA Medical College, Beijing, China

**Keywords:** Aβ, Alzheimer’s disease, tau, NFTs, biomarkers

## Abstract

Alzheimer’s disease (AD) is a progressive degenerative neurological illness with insidious onset. Due to the complexity of the pathogenesis of AD and different pathological changes, the clinical phenotypes of dementia are diverse, and these pathological changes also interact with each other. Therefore, it is of great significance to search for biomarkers that can diagnose these pathological changes to improve the ability to monitor the course of disease and treat the disease. The pathological mechanism hypothesis with high recognition of AD mainly includes the accumulation of β-amyloid (Aβ) around neurons and hyperphosphorylation of tau protein, which results in the development of neuronal fiber tangles (NFTs) and mitochondrial dysfunction. AD is an irreversible disease; currently, there is no clinical cure or delay in the disease process of drugs, and there is a lack of effective early clinical diagnosis methods. AD patients, often in the dementia stages and moderate cognitive impairment, will seek medical treatment. Biomarkers can help diagnose the presence or absence of specific diseases and their pathological processes, so early screening and diagnosis are crucial for the prevention and therapy of AD in clinical practice. β-amyloid deposition (A), tau pathology (T), and neurodegeneration/neuronal damage (N), also known as the AT (N) biomarkers system, are widely validated core humoral markers for the diagnosis of AD. In this paper, the pathogenesis of AD related to AT (N) and the current research status of cerebrospinal fluid (CSF) and blood related biomarkers were reviewed. At the same time, the limitations of humoral markers in the diagnosis of AD were also discussed, and the future development of humoral markers for AD was prospected. In addition, the contents related to mitochondrial dysfunction, prion virology and intestinal microbiome related to AD are also described, so as to understand the pathogenesis of AD in many aspects and dimensions, so as to evaluate the pathological changes related to AD more comprehensively and accurately.

## Introduction

Alzheimer’s disease (AD), commonly known as senile dementia, is defined as a chronic, progressive developmental neurodegenerative disease caused by multiple factors and characterized by diffuse cerebral cortical atrophy (Andrade-Guerrero et al., 2023). The major clinical symptoms are reduced judgment ability and memory impairment, accompanied by aphasia, miscalculation, agnosia and other characteristics (Chu and Liu, 2019; Guo et al., 2021; Andrade-Guerrero et al., 2023). The characteristic pathological changes mainly include β-amyloid (Aβ) deposits forming senile plaques, abnormally phosphorylated intracellular tau protein contributing to neuronal fiber tangles (NFTs), and a reduction in neuronal cells (Chu and Liu, 2019; Guo et al., 2021; Wu et al., 2021; Zhang et al., 2021; Hao et al., 2022).

Alzheimer’s disease, along with malignant tumors and cardiovascular diseases, is one of the primary three causes of death for seniors (Zhang et al., 2021). As the demographic structure of our society ages, AD is becoming a major medical and health problem in our society today due to its increasing frequency and incidence (Wu et al., 2021; Zhang et al., 2021). China will become the region with the largest and fastest-growing AD population in the world (Mamun et al., 2020; Wu et al., 2021; Zhang et al., 2021; Hao et al., 2022). However, there is still a lack of effective drug treatment, and delayed intervention timing may be an important reason for poor drug trial results (Karceski and Antonopoulos, 2023). If the disease can be diagnosed and treated before symptoms or irreversible pathology appears, unexpected results may be achieved. It is currently believed that AD is a continuous disease process from pathological changes to clinical symptoms, which is divided into three stages: preclinical AD, mild cognitive impairment (MCI) and AD (Karceski and Antonopoulos, 2023). Each stage has corresponding biomarkers as diagnostic criteria, so it is of great significance to carry out screening in the early stage of mild cognitive impairment in AD disease. The study of molecular pathophysiological mechanisms and the development of biomarkers at different stages of the progression of AD will lay the foundation for more accurate and personalized biomarker targeted therapy and further promote precision medicine. According to classical theory, the pathological process of AD mainly includes the loss of neurons, the formation of neurofibrillary tangles and Aβ in nerve cells (Wu et al., 2021, 2022). As the most important biomarker for AD, Aβ has been extensively studied, and several studies have shown that the AD biomarker in CSF can be detected before the MCI stage, and the Aβ42 in cerebrospinal fluid (CSF) in AD patients is 50% lower than that in normal people, and the Aβ42/Aβ40 ratio is closer to the clinical data (Ritchie et al., 2017). However, the acquisition of CSF is relatively harmful to the human body, so blood biomarkers that are easy to obtain have gradually been favored by people (Frank et al., 2022). Studies have shown that blood Aβ42 levels also decline with cognitive decline in patients, and the changes in the blood Aβ42/Aβ40 ratio are consistent with the changes in CSF (McGrowder et al., 2021). Tau begins to accumulate during the MCI stage, so it cannot be used as a predictor of AD, only as a diagnosis. Elevated t-tau protein is associated with a variety of neurodegenerative diseases, so it cannot be used as a specific marker of AD (McGrowder et al., 2021). P-tau181 has been reported to be characteristically elevated in CSF and therefore can be used as a specific biomarker of CSF for AD (Giacomucci et al., 2022). The researchers found that p-tau217 in plasma was more accurate than p-tau181 in distinguishing between AD and other neurodegenerative diseases, but it is not yet in clinical use (Frank et al., 2022; Giacomucci et al., 2022).

Aβ42, Aβ42/Aβ40 and p-tau in body fluids are the established core biomarkers, and other molecular pathological biomarkers have also made significant progress in the study of AD markers. Next, this paper discusses the application of biomarkers for AD and the progress of related pathogenesis research.

## Beta-amyloid cascade hypothesis

### Beta-amyloid protein and Alzheimer’s disease

The amyloid cascade hypothesis proposes that excessive production or untimely clearance of Aβ42 or other Aβ polypeptide fragments leads to the deposition of soluble Aβ oligomers and insoluble amyloid in the brain to form amyloid plaques and post-translational modifications (PTMs) can enhance this phenomenon by altering the structure of Aβ polypeptide chains (Roher et al., 2017; Zhou et al., 2022). The interactions between Aβ and microglia, astrocytes, vasculature and neurons trigger a number of deleterious cellular reactions that result in neuronal malfunction and death, ultimately leading to AD (Roher et al., 2017; Yang Y. et al., 2022; Zhou et al., 2022). The amyloid cascade hypothesis has been proposed for more than 30 years and is now the most widely accepted hypothesis to explain the relevance of AD, but no satisfactory results have been achieved in the last decade for Alzheimer’s-related drugs targeting Aβ (Sala Frigerio and De Strooper, 2016; Ritchie et al., 2017; Pan et al., 2018). Therefore, the role of Aβ in AD is highly controversial, and some researchers believe that the development and progression of AD are significantly impacted by soluble Aβ or that Aβ is not the only causative protein (Ritchie et al., 2017; Wu et al., 2022).

### The production of beta-amyloid

Aβ is a protein molecule of 39–43 amino acid residues produced by the hydrolysis of Aβ precursor protein (APP) by the enzyme complex α, β and γ-secretase and is called Aβ because of its β-folding in the three-dimensional spatial structure (Wu et al., 2022). Continuous amyloid production leads to the formation of high molecular weight insoluble Aβ fibers, which are deposited to form age spots (Zhao et al., 2014; Wu et al., 2022). The *APP* gene on chromosome 21 encodes APP. The function of APP can be summarized as “neurotrophic support” and supporting healthy neuronal function, or “amyloidosis” and pathological effects in the brain (Zhao et al., 2014). APP is a glycosylated intact transmembrane protein with approximately 695–770 amino acids, which is acted upon by a series of splitting enzymes to produce a series of neuroactive peptides, of which the βAPP signaling pathway is via neurotrophic, amyloidogenic or phagocytic (clearance) pathways (Zhao et al., 2014; Wang et al., 2017). It has a larger N-terminal extracellular domain and a smaller C-terminal cytoplasmic domain (Wang et al., 2017). And the *Swedish* mutation is familial Alzheimer’s disease (FAD) associated with the *APP* mutation. It has been shown that the *Swedish* mutation causes the production of β-secretase to exceed that of γ-recretase, resulting in more Aβ production (Szaruga et al., 2017; Zhou et al., 2022).

Enzyme digestion takes two different paths: the non-amyloid pathway, which provides beneficial neuro-nutrients, and the amyloid pathway, which produces neurotoxic Aβ peptides, which then misfold and aggregate to form deposits that can cause AD (Ritchie et al., 2017; Wu et al., 2022).

**FIGURE 1:**
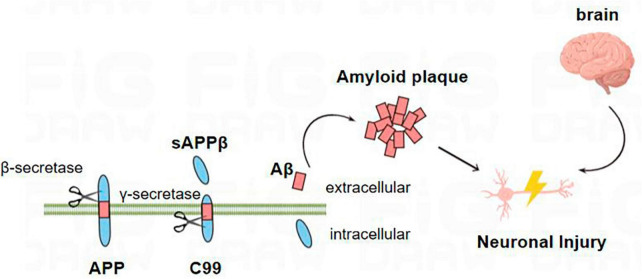
Amyloid metabolic pathway: the N-terminal extracellular domain portion of APP is first cleaved by β-secretase to produce soluble sAPPβ fragments and C99. C99 is further processed by β-secretory enzymes at multiple sites to release Aβ. Aβ proteins aggregate into oligomers and fibrils and eventually form plaques that damage neurons, leading to the occurrence of AD.

In the amyloid metabolic pathway, the N-terminal extracellular domain portion of APP is first cleaved by β-secretase, producing the soluble sAPPβ fragment and the CTFβ (C99) complex (Wu et al., 2022). The sAPPβ fragment is released into the extracellular matrix, and C99 is membrane-bound to the 99 amino acids at the C-terminal of APP (Takasugi et al., 2023). Multiple sites of γ-secretase’s subsequent processing of C99 result in the discharge of the Aβ42 (Figure 1; Roher et al., 2017; Suzuki et al., 2023; Takasugi et al., 2023; Wilkins, 2023). Aβ40 contains 40 amino acids, and Aβ42 contains 42 amino acids. The basic chemical properties of polypeptides depend on the type of amino acid residues contained, and it is generally believed that Aβ42 has relatively strong neurotoxic effects because the 38th–42nd amino acids at the C-terminal of the Aβ peptide are non-polar acids, which reduce the solubility of the polypeptide chain and increase the aggregation tendency (Roher et al., 2017).

The production of Aβ42 is considered to be the driving factor leading to the development of AD, but in the study of Szaruga et al. (2017), the production of longer Aβ peptides may be highly correlated with the development of AD (Zhou et al., 2022). γ-Secretase cleavage of APP can produce different lengths of Aβ peptide, while AD-induced *PSEN* mutations continuously reduce γ-secretase activity, making the γ-secretase and APP complex unstable and easy to dissociate, resulting in longer and more Aβ amyloid peptide (Rajendran et al., 2006). Peter S. Klein found that lithium, an inhibitor of Glycogen Synthase Kinase-3 (GSK-3), blocks Aβ peptide production by interfering with the γ-secretase cleavage step. The authors then found by RNA interference that the expression levels of Aβ42 and Aβ40 were significantly decreased after the expression of GSK-3α was decreased, and overexpression of GSK-3α in CHO-APP695 cells could increase the levels of Aβ40 and Aβ42 (Roher et al., 2017). However, the decreased expression of GSK-3β increased the expression of Aβ40 and Aβ42. GSK-3α and GSK-3β share 70% of the same kinase domain, and the carboxyl and amino terminal sequences are different, which may lead to different processing regulation of APP (Phiel et al., 2003).

In addition to the amyloid production pathway, there is also a non-amyloid production pathway during APP metabolism. The non-amyloid degradation pathway is also divided into two stages of cleavage (Roher et al., 2017). The second-stage cleavage enzyme is also γ-secretase, while the first-stage shearing enzyme is α-secretase (Roher et al., 2017; Zhou et al., 2022; Takasugi et al., 2023). α-Secretase is cleaved between residues 16 Lys and 17 Leu of the intracellular portion of Aβ on APP, and the resulting 3 kDa fragment is a fragment of sAPPα polypeptide that lacks 16 amino acids in the N-terminal of Aβ (Zhou et al., 2022). The soluble Aβ peptide fragment with the N-terminal of Aβ lacks 16 amino acids (Brinkmalm and Zetterberg, 2021; Wu et al., 2022). The sAPPα polypeptide produced by the non-amyloid pathway has various roles, such as protecting neurons from oxidative stress, guiding neuronal development, improving synaptic function, and monitoring neuroactive compounds and pathogens (Phiel et al., 2003). In addition to Aβ, other neurotoxic peptide chains produced by APP hydrolysis may play a crucial role in the development of late-onset AD (Pan et al., 2018).

Aβ can also be found in the CSF of healthy humans. It is not a product of lesions produced in the brains of patients with AD but a molecule produced and secreted during the normal metabolism of APP. The production and degradation of Aβ under normal conditions are in dynamic balance (Kent et al., 2020; Zhou et al., 2022). Under pathological conditions, Aβ can have strong neurotoxic effects after precipitating and aggregating in the cytoplasmic matrix (Tolar et al., 2020). The aggregation of Aβ (whether oligomers or age spots) leads to the disturbance of cellular calcium signaling, affects mitochondrial oxidative stress, energy metabolism and glucose metabolism, and leads to the loss of synapses and neuronal death, as well as the occurrence of a series of neuroinflammation, which is the Aβ hypothesis that currently dominates the pathogenesis of AD (Sala Frigerio and De Strooper, 2016; Tolar et al., 2020). Other studies suggest that Aβ is a by-product of AD and has a protective effect on neurons in the early stage of AD (Sala Frigerio and De Strooper, 2016).

Usually, a certain level of Aβ in the brain can be maintained by rapidly clearing the Aβ produced in the brain. Studies have shown that both impaired production and clearance of Aβ can disrupt the balance of Aβ in the brain, causing AD. Increased production of Aβ is more common in FAD and has been shown to be connected to mutations in the *PS1*, *PS2*, and *APP* genes (Szaruga et al., 2017; Zhou et al., 2022; Suzuki et al., 2023). Most pathogenic mutations in the *APP* gene occur at the BACE1 cleavage site of APP, the γ-secretase cleavage site, or near the Aβ sequence, leading to increased Aβ42, the Aβ42/Aβ40 content ratio, and total Aβ levels by increasing enzymatic cleavage efficiency (Roher et al., 2017). Mutations in the *PS1* and *PS2* genes enhance γ-secretase activity and increase the Aβ42/Aβ40 content ratio (Zhao et al., 2014). Mutations produce Aβ that is deposited in the brain and becomes toxic to neurons. Neurotoxic Aβs are produced more frequently as a result of *APP* mutations that interfere with the cleavage reaction. Suzuki et al. (2023) used a two-yeast hybridization system and found that two mutations of *APP*, including proline and aspartic acid residues, enhanced the cleavage reaction of γ-secretase to APP. The proline M722D mutation mimicked the FAD mutation, reducing the production of Aβ40 and increasing the production of Aβ42 (McGrowder et al., 2021). The authors found by NMR waves and electron microscopy that the transmembrane part of APP’s α-helix and β-sheet structures are made more flexible and susceptible to ε-cleavage by proline alterations; by strengthening the association with the positively charged PS1 K380 residue, aspartate facilitates APP cleavage (Suzuki et al., 2023). Szaruga et al. (2017) used a progressive temperature increase method to demonstrate that wild-type γ-secretase production of the “disease-like” Aβ peptide is induced with increasing temperature. More and longer Aβ amyloid peptides are produced as a result of the decreased activity of γ-secretase caused by a rise in temperature. The author found that both the pathogenic *PSEN1* mutation and the increase in temperature resulted in the production of longer Aβ peptides by reducing the catalytic efficiency of the enzyme on the substrate, rather than reducing the affinity of the enzyme to the substrate (Rajendran et al., 2006). Other studies have also shown that the CpG site in the promoter region of the *APP* gene is hypermethylated in the AD brain, causing *APP* gene overexpression to result in increased Aβ production. Recently, some researchers reported their team used CRISPR gene-editing technology to reduce amyloid deposition, and their goal was to cut out a small portion of the C-terminal of APP protein using CRISPR gene-editing technology, enhancing levels of sAPPα while preventing APP from being cut by the β-secreting enzyme (Bhardwaj et al., 2022).

The occurrence of AD also involves multiple impaired Aβ clearance mechanisms. The clearance of Aβ under physiological conditions involves both extracellular and intracellular pathways. The intracellular pathway includes the autophagy-lysosome and ubiquitin-proteasome systems, while the extracellular pathway includes protease degradation, microglia phagocytosis, and the translocation of Aβ from tissue fluid and cerebrospinal fluid to the cervical lymph nodes, peripheral blood, and liver and kidney for clearance (Fang et al., 2019). The intracellular pathway plays a role in removing misfolded proteins as well as preventing the accumulation of abnormal proteins (Yang L. et al., 2022). Misfolded Aβ in AD is not easily degraded, directly impairing both the intracellular pathway and intensifying the accumulation of Aβ. A variety of AD risk factors, including stroke and hypertension, can result in blood-brain barrier (BBB) dysfunction, which decreases the amount of intracranial Aβ transported across the BBB to the periphery (Ashleigh et al., 2023). BBB dysfunction can also trigger neuroinflammation, oxidative stress, and enhanced BACE1 and γ-secretase activity, all of which are associated with Aβ accumulation. In Dale Schenk’s study, immunotherapy of PD mice with amyloid plaque-related protein was used. Compared with the control group, non-amyloid deposition was found in the brain sections of mice injected with Aβ42 immune protein, and astrocytes were correspondingly reduced. A large amount of β-amyloid deposition was found in the hippocampal tissue of the control group. It contains a large number of clusters of astrocytes. This suggests that the immune response against the plaque components themselves contributes to the elimination of beta-amyloid plaques and pathological reactions (Schenk et al., 1999).

Clearing the brain of neurotoxic Aβ has been the main therapeutic strategy for the treatment of AD (Yang L. et al., 2022). However, there are many adverse reactions and inconveniences in the intervention of direct elimination of Aβ in the central system. Scientists have developmented a novel approach to promote central Aβ outflow by peripheral clearance of Aβ, and then indirectly reduce the deposition of Aβ in the brain to improve related neuropathological changes and cognitive function (Yang L. et al., 2022). This study has found the mechanism of regulating the clearance of Aβ by peripheral macrophages, thus providing A new idea for the effective treatment of Alzheimer’s disease (Yang L. et al., 2022).

### Pathological mechanism of Aβ

Microglia has a double-edged sword effect on AD; on the one hand, they can release inflammatory factors and trigger an inflammatory response; on the other hand, in the pre-disease stage of AD, microglia can phagocytose abnormally deposited Aβ, and the accumulation of Aβ impairs the phagocytic function of microglia and promotes the release of inflammatory factors (Zhao et al., 2014; Zhang et al., 2021). It has been shown that microglia can promote the reproduction of Aβ, and Yang L. et al. (2022) demonstrated that microglia clearance significantly reduced intracellular Aβ deposition using *Irf8-/-/Cx3cr1* ± transgenic mice. After treating microglial cells with Aβ1-42, Hao et al. (2022) detected a significant decrease in the level of the lysosome marker Lmap1, indicating that Aβ can induce lysosomal damage in microglial cells and reduce Aβ clearance. Subsequent treatment with MExo-Gem restored the ability of microglia to clear Aβ. In October 2022, John R. Lukens published an article in cell showing that SYK regulates the activation and function of microglia, and that impaired function is associated with the deterioration of neurodegenerative disease models such as AD and multiple sclerosis (Ennerfelt and Lukens, 2023). In AD model mice, microglial SYK loss leads to increased Aβ deposition, neuropathological aggravation, and cognitive deficits. Loss of SYK signaling impedes the development of disease-associated microglia, alters AKT/GSK3β signaling, and limits microglia phagocytosis (Ennerfelt and Lukens, 2023).

Acetylcholine (ACh) is believed to be involved in learning and memory, and studies have shown that Aβ can cause acetylcholinergic nerve damage by promoting the release of choline from neurons into the cell, depleting the intracellular choline, and thus reducing ACh synthesis (Akıncıoğlu and Gülçin, 2020); Aβ can also activate tau protein kinase1, which phosphorylates pyruvate dehydrogenase, thereby reducing the conversion of pyruvate to acetyl-CoA, which is the raw material for the synthesis of ACh, thereby reducing the synthesis of ACh. Aβ promotes the opening of potassium ion channels in cholinergic nerve membranes, promotes K^+^ outflow, and leads to cell death (Saxena and Dubey, 2019; Akıncıoğlu and Gülçin, 2020).

Aβ peptide can induce oxidative stress, which can be regarded as the coupling molecule between oxidative stress and AD brain cell death, and lead to oxidative stress through various ways: Aβ itself can act as oxygen free radical donor and produce reactive oxygen species; Aβ can also activate microglia by binding to specific receptors, while producing A large number of free radicals, exacerbating oxidative stress (Wang et al., 2005; Pan et al., 2018). Decreased local cerebral perfusion is one of the early clinical manifestations in sporadic and familial AD patients. Most AD cases have been found to have Aβ deposition in cerebral blood vessels leading to cerebral amyloid vasculitis and hemorrhagic shock (Pan et al., 2018).

β-Secretase activity is positively correlated with age, and the contents of C99 and Aβ are increased after BACE1 is activated. C99 accumulates significantly in *APP* and *PS1* mutant FAD patients (Takasugi et al., 2023). The fact that C99 is not easily degraded, that accumulated C99 disrupts the intracellular lysosomal degradation pathway and that accumulated C99 saturates the substrate binding site of γ-secretase suggests that C99 may also have a link to the pathogenesis of AD (Takasugi et al., 2023). C99 damages mtDNA, induces the release of cytochrome C, and causes apoptosis.

In conclusion, the neurotoxicity of Aβ is a complex process with multiple pathways and resourcefulness. For example, abnormal immune function and super-strong inflammatory response, oxygen free radicals interact and promote each other, causing cell structure destruction and even necrosis, or inducing cell apoptosis, and eventually leading to the characteristic pathological changes and progressive cognitive dysfunction of AD.

## Alzheimer’s disease-related tau pathological changes

### Structure and function of tau

Another pathological characteristic of AD patients is the hyperphosphorylation of tau proteins in the brain, leading to excessive deposition of some amyloid proteins in the brain tissue, leading to the development of specific senile plaques and causing some neuronal tangles leading to neuronal damage and further affecting the function of the neuronal system in the patient’s brain (Wu et al., 2021). The *MAPT* gene encodes tau proteins. Due to the different ways of tau mRNA editing, 6 kinds of isomers can be expressed in normal people (Wu et al., 2021). The six subtypes share extensive functional similarities but have unique physiological roles. The binding rates of the six subtypes of tau protein to microtubules are also very different (Ittner et al., 2016). In several investigations, ON4R was found to be more common among the six isoforms in AD, whereas 2N3R and 1N3R are markedly diminished in AD patients (Wang et al., 2017). Tau is primarily produced by glial and neuronal cells in the central nervous system. Tau proteins are divided into four regions: the C-terminal region, microtubule-binding structural domain, proline-rich structural domain, and N-terminal region (Wu et al., 2021).

The microtubule system is a cytoskeletal component of nerve cell and is involved in a variety of cellular functions. Microtubules are composed of tubule protein and tubule associated protein, and tau protein is the most abundant microtubule associated protein. In normal neurons, tau protein is mainly enriched around neuronal axons (Wu et al., 2021). As the “scaffold” of microtubules, tau protein participates in microtubule assembly and acts on the distal end of axons to maintain microtubule stability and flexibility; tau proteins are also involved in regulating axonal transport and protecting the integrity of DNA in the nucleus (Tracy et al., 2022).

### Hyperphosphorylation of tau protein

Post-translational modifications are important modalities that regulate protein structure and function. Tau proteins have various forms of PTMs, such as phosphorylation, glycosylation and acetylation (Naseri et al., 2019). Under normal physiological conditions, these modifications are widespread, but under pathological conditions, the phosphorylation of tau protein increases to 2–3 times the normal level. Tau phosphorylation at different sites reflects the course of the disease (Chu and Liu, 2019). And the degree of tau protein phosphorylation is negatively correlated with its involvement in microtubule assembly. Both hyperphorylation and aggregation of tau protein lead to microtubule dysfunction and impaired axonal transport and result in abnormal mitochondrial distribution (Ittner et al., 2016).

*In vivo*, tau proteins are phosphorylated by kinases such as CDK5, GSK-3β, MAPK, and PKA. In the AD brain, after the tau protein is over phosphorylated by GSK-3β, CDK5, and other phosphorylated kinases, p-tau protein has 10% of the normal tau protein’s binding force when it binds to microtubule protein, and tau protein is detached from microtubules (Phiel et al., 2003); it also misses its biological role of retaining microtubule stability and boosting microtubule formation (Pan et al., 2018). Studies have shown that inflammatory processes in the aging brain lead to phosphorylation of tau proteins in common late-onset AD. “We are able to reduce tau phosphorylation by restoring the regulatory effects that are lost with age and inflammation,” said Arnsten (Bathla et al., 2023). Members of Arnsten’s lab investigated ways to reduce tau phosphorylation early in disease progression, before neurons are damaged. Specifically, they focused on the role of GCPll (glutamate carboxypeptidase-II), a brain enzyme involved in inflammation (Bathla et al., 2023). This enzyme weakens the protective effect provided by mGluR3 (metabolized glutamate receptor 3), a receptor on neurons that promotes higher cognitive function. In their research, members of Arnsten’s lab investigated ways to reduce tau phosphorylation early in disease progression (before neuronal damage) (Bathla et al., 2023). Specifically, they focused on the role of GCPll (glutamate carboxypeptidase-II), a brain enzyme involved in inflammation. This enzyme weakens the protective effect provided by mGluR3 (metabolized glutamate receptor 3), a receptor on neurons that promotes higher cognitive function (Bathla et al., 2023).

### Paired helical filaments

Two highly phosphorylated tau monomers can combine to form a dimer due to structural changes (Ittner et al., 2016). This dimerization begins with the interaction of hexapeptides at repeat sites 2 and 3, which then leads to the oligomerization of tau proteins. The oligomers further form paired helical filaments (PHFs) with a double helix bond structure (Ittner et al., 2016). Tau proteins in PHFs have more negative charge than tau monomers and are therefore unable to bind to negatively charged tubules or effectively stabilize microtubules. Without tau’s stability, the network of microtubule structures easily disintegrates, causing neurons to no longer function properly (Ittner et al., 2016; Fišar, 2022; Denechaud et al., 2023).

### Neurofibrillary tangles

In the early stages of AD, NFTs are not yet formed, and phosphorylation of tau protein occurs mainly at Ser199 and Ser422, with phosphorylation at Ser202 and Thr205 increasing as the disease progresses (Denechaud et al., 2023). Phosphorylation at Thr231 generally marks the formation of more mature p-tau assembly NFTs, driving the disease to an advanced stage. The extent to which NFTs are deposited in the brain is associated with the severity of dementia and neuronal death (Frank et al., 2022; Giacomucci et al., 2022). Other forms of tau aggregates in AD patients, including nerve fiber webs and nerve plaques, also cause neuronal decline. While some studies have suggested that filamentous and fibrous tau may have protective effects on neurons, highly phosphorylated tau oligomers are the most toxic type (Frank et al., 2022).

### Pathological changes in tau

**FIGURE 2:**
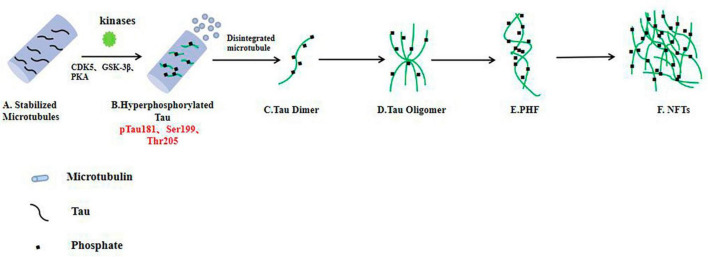
In the AD brain, hyperphosphorylated tau protein disrupts the stability of neuronal microtubules and forms NFTs, resulting in impaired neuronal function. **(A)** Tau proteins bind to microtubule binding domains to stabilize microtubules. **(B–F)** In the brain neurons of AD patients, phosphorylated kinases such as CDK5 and GSK-3β overphosphorylated tau protein, hyperphosphorylated tau protein separated from microtubules, and the microtubule depolymerized to produce insoluble tau oligomers, tau monomers aggregated to form oligomers, which then intertwine with each other to form pairs of spiral filaments, eventually forming NFTs.

In all age-related neurodegenerative diseases, the progressive decline in neuronal function and loss of neurons (loss of synapses, dendrite pruning, and reduced neuronal repair and recovery) is the key biological basis and the most appropriate and powerful neurobiological event to explain clinical dementia-cognitive impairment (Müller et al., 2017; Chu and Liu, 2019; Denechaud et al., 2023). Synapses allow normal brain function to communicate, and synaptic loss has the strongest correlation with cognitive decline. Neurons require a constant supply of energy, most of which is used for synaptic transmission (Cheng and Bai, 2018; Chu and Liu, 2019; Frank et al., 2022). As mentioned above, over phosphorylated tau protein aggregates around cells to form PHFs, which aggregate to form NFTs and eventually lead to synaptic dysfunction (Figure 2). Phosphorylation of the tau protein was found to be attenuated by the use of a GSK-3β phosphorylation inhibitor, which ameliorated synaptic damage in *APP/PS1* transgenic mice (Brinkmalm and Zetterberg, 2021). PHF-Tau competes with microtubule proteins to bind normal tau proteins and other large microtubule-associated proteins and removes these proteins from microtubules, which causes the depolymerization of the microtubules and disturbance of the normal vascular system, while abnormally phosphorylated tau proteins then aggregate themselves into PHF/NFT structures (Fišar, 2022). Extensive disruption of neuronal microtubule structures in the brains of AD patients and impaired normal axonal transport cause synaptic loss, neuronal functional impairment and cerebral neurodegenerative lesions. It has been shown that knocking down the expression of tau protein in primary neurons destabilizes microtubules, affects neuronal growth and delays neuronal maturation (Kent et al., 2020); however, abnormal expression of tau protein does not alter axonal transport kinetics.

Researchers have found that tau proteins accumulate at synaptic terminals in the hippocampus and internal olfactory cortex of AD patients and promote impairments in synaptic plasticity (Chu and Liu, 2019; Kent et al., 2020; Denechaud et al., 2023). Similarly, NFT has been associated with synaptic dysfunction and cognitive decline (Chu and Liu, 2019). Previous studies have shown that tau proteins can be transmitted through synaptic secondary neurons in model rats. The uptake of tau protein in the form of oligomers outside the cell by neurons triggers the accumulation of tau protein inside the cell, leading to the formation of new tau tangles (Tracy et al., 2022). This impairs axonal transport and affects the function of neurons. This suggests that targeting tau proliferation may be a potential treatment strategy for AD.

Tau proteins bind to presynaptic proteins and inhibit the release of prominent vesicles, thereby blocking neurotransmitter transmission (Wu et al., 2021; Tracy et al., 2022). Reducing presynaptic protein expression and restoring neurotransmitter release may alleviate synaptic dysfunction in AD. Tara E. Tracy used the Engineered Ascorbate Peroxidase (APEX, an effective method for studying protein-protein interactions in living cells) method to detect the interaction of the N-terminal of tau protein with synaptoprotein-3, inhibiting the release of presynaptic vesicles (Tracy et al., 2022). Phosphorylated tau protein is protective of synaptic terminals and memory deficits in mice. Phosphorylated tau proteins are protective of synaptic terminals, and researchers found that p38 γ-mediated phosphorylation of tau proteins in *APP23* mice inhibited Aβ-induced excitotoxicity (Ittner et al., 2016). Ittner et al. (2016) found that phosphorylation of tau at Thr205 by p38γ inhibits the neurotoxic effects of Aβ in the early stages of the disease. Arne Ittner crossed *p38*γ ^–/–^ mice with AD model *APP23* mice and found that loss of p38γ increased excitotoxicity, memory deficits, and premature death in *APP23* mice (Ittner et al., 2016). In AD patients and *APP23* mice, the authors found that p38γ levels decreased significantly, and compared to controls, p38γ-expressing neurons demonstrated a considerable increase in resistance to Aβ-induced cell death (Ittner et al., 2016).

Excessive Tau protein in neurons has long been considered a risk factor for AD (Naseri et al., 2019), however recent research has challenged this view (Denechaud et al., 2023). The research team found that neurons in the brains of AD patients are activated to re-enter the active state of cell cycle after being damaged by oxidative stress (normal neurons are at rest), and at this time, more dephosphorylated tau protein will accumulate in the nucleus of neurons, limiting the cells in the S phase, thus preventing neurons from passing the S phase and apoptosis (Denechaud et al., 2023). It has certain protective effect on neurons. This result suggests that tau is not necessarily a killer, and may even play the role of protector at some times, giving us a deeper understanding of tau’s complex role in the pathological changes of AD (Denechaud et al., 2023).

The trigger receptor (TREM2) expressed on bone marrow cell 2 is a transmembrane protein that is primarily expressed by microglia in the brain (Zhang et al., 2023). Several studies have found that silencing of TREM2 in the brain exacerbates tau pathology, while overexpression of TREM2 improves tau pathology, but other studies have found that loss of TREM2 does not affect tau phosphorylation, while TREM2 activation exacerbates tau pathology (Zhang et al., 2023). Recently, it has been found that sTREM2 selectively binds to TG2 [transgelin-2 (TG2) expressed on neurons as a receptor for sTREM2], inhibits downstream RhoA-ROCK pathway, and attenuates tau hyperphosphorylation (Zhang et al., 2023). Overexpression of *sTREM2* attenuated tau pathology and cognitive impairment in tau *P301S* transgenic mice. In addition, sTREM2 (Laske et al., 2017; Hampel et al., 2018; Hansson et al., 2018; Lleo et al., 2019; Doecke et al., 2020; Aamodt et al., 2021; Clark et al., 2021; Simrén et al., 2021; Park et al., 2022; Zhang et al., 2022; Delaby et al., 2023; Dinoto et al., 2023; Pena-Bautista et al., 2023) is the smallest sTREM2 sequence that activates TG2 (Zhang et al., 2023). This active peptide mimics the protective effects of sTREM2 both *in vitro* and *in vivo*. To sum up, the study found that microglia secrete sTREM2, which may play a protective role in tau pathology.

Several treatment research options for AD and other tau diseases involve targeting the protein. Tau protein hyperphosphorylation is regulated by protein kinases and phosphatases, and treatment for tau protein hyperphosphorylation requires inhibition of tau kinase, repair of protein phosphatase, or o-glycosylation of tau protein. In addition, the treatment of neuroinflammation is also a research direction, because neuroinflammation also promotes the proliferation of tau.

### Prion biology

Most age-related neurodegenerative diseases, including AD, occur with the misfolding and aggregation of specific proteins within the nervous system (Condello et al., 2020; Flach et al., 2022). Such diseases are called protein misfolding diseases or proteinopathies and share similar biophysical and biochemical characteristics to prion diseases. In AD, Aβ and tau proteins are called “prion-like molecules,” indicating that they have prion-like biological properties (Flach et al., 2022). Aβ and tau protein are induced to misfold by a templated structural change. The misfolded molecules can not only further influence the normal molecules to make their structural changes, but also easily aggregate to form a subset aggregator (Hooper and Turner, 2008; Di Guardo, 2018; Li et al., 2021). Such abnormal molecular aggregates can cause lesions in the local parts of the brain tissue, and through the cellular transmission of the subaggregates, the tissue damage is also dispersed far (Flach et al., 2022; Zattoni et al., 2022).

A prion is not a virus, but a disease causing agent made of proteins. It does not contain nucleic acids, but can replicate itself and is infectious. The pathogen can cross the blood-brain barrier, slowly destroying the structure of brain tissue and eventually killing the patient (Di Guardo, 2018; Flach et al., 2022). Alzheimer’s disease is a “diprion” disease in which Aβ and tau tangles in a patient’s brain can infect healthy brain tissue just like prion proteins, only this time at a much slower rate (Kent et al., 2020). Professor Diamond found that self-reproducing prion forms of tau and Aβ were most infectious in the brains of patients who died at a young age from the inherited gene-driven form of AD, but were less common in the brains of patients who died at an older age (Aoyagi et al., 2019). The researchers found an extremely strong correlation between tau prions in particular and the age at which the patients died: relative to overall tau levels, the amount of tau prions in the brains of patients who died at age 40 was, on average, 32 times higher than that of patients who died at age 90 (Aoyagi et al., 2019). The misfolding of proteins causes the cellular mechanisms for clearing them to be ineffective. Mishandled by this particular protein, which determines the dysfunction of different groups of neurons and thus leads to the clinical manifestations of the disease, leading to tissue damage and cell necrosis (Fang et al., 2019; Karimian et al., 2020; Kent et al., 2020; Tolar et al., 2020; Bhardwaj et al., 2022; Ashleigh et al., 2023).

Shafiq et al. (2021) isolated prion proteins from the brains of AD patients using sucrose density gradient centrifugation and then concluded by co-immunoprecipitation binding mass spectrometry and other methods that prion proteins bind to the tubulin/actin of neurons, leading to neuronal cytoskeleton damage and disintegration (Schenk et al., 1999). Normally, tau protein usually exists in the form of natural monomers that can be detected in the synapses of neurons. However, in the brains of AD patients, tau protein is converted from the inactive form to the active “seed” form, and tau proteins continuously form and overlap, forming a large number of oligomers or neurofilament tangles (Hao et al., 2022; Takasugi et al., 2023). Tau protein oligomers have prion-like transmissibility, and hyperphosphorylated tau protein can isolate normal tau protein into oligomers, which then spread across cells between neurons (Mamun et al., 2020). According to reports, tau protein in the cerebral cortex begins in the medial temporal lobe and subsequently diffuses to the ventral and lateral adjacent areas of the temporal lobe, retrosplenial cortex and posterior cingulate (Mamun et al., 2020). In the brains of AD patients, tau protein spreads more extensively, affecting the neocortex of the brain. Autopsy results demonstrate that the accumulation of NFTs is associated with cognitive deficits and neurodegenerative diseases (Lleo et al., 2019).

In 2021, scientists discovered the mechanism by which microglia promote the pathological spread of Aβ into healthy brain tissue (Yang L. et al., 2022). The researchers transplanted the embryonic nerve cells of WT mice into the 5xFAD cortex of AD mice, and found that Aβ plaques appeared in the transplanted WT tissues after 4 weeks, and gradually increased over time (Yang L. et al., 2022). They then found that microglia engulf Aβ from the tissue *in situ* and migrate into the tissue, resulting in Aβ deposition in WT tissue (Yang L. et al., 2022). Intervention in the phagocytosis and migration of microglia can improve the pathological deposition of Aβ, so targeting the function of microglia may be a powerful means to solve the proliferation of Aβ.

## Mitochondrial dysfunction and autophagy

Mitochondria play an important role in apoptosis, anti-oxidation and energy production. Neurons are cells with high energy demand, so they are highly dependent on mitochondria (Ashleigh et al., 2023). Under physiological conditions, mitochondrial biogenesis, fusion, fission and autophagy work together to maintain mitochondrial homeostasis. However, in some cases, mitochondrial dysfunction can lead to a range of consequences such as reduced ATP production and apoptosis (Cheng and Bai, 2018). In neurodegenerative diseases, apoptosis is a genetically determined programmed cell death that is an important way of mediating neuronal cell death. When apoptosis is abnormally increased, it can lead to neurodegenerative diseases (Li et al., 2022; Ashleigh et al., 2023). Apoptosis is divided into extrinsic and intrinsic pathways (also known as mitochondrial pathway, which is the main apoptotic pathway in AD disease, and cytochrome C plays a major role). With the development of AD disease, the reduction of ATP synthesis and the increase of reactive oxygen species (ROS) induce apoptosis, and mitochondrial dysfunction will release pro-apoptotic factors. Binding to cytoplasmic factors produces apoptotic bodies that either directly activate or indirectly trigger apoptosis (Cheng and Bai, 2018; Dhapola et al., 2022; Fišar, 2022).

Mitochondria are highly dynamic, complex organelles that primarily provide energy, but also play key roles in cell death, signaling pathways, apoptosis, ROS production, and calcium homeostasis, all of which are disrupted in neurodegeneration (Fišar, 2022; Li et al., 2022). The brain is one of the highly metabolic organs, and neurons strongly rely on mitochondria to function and are susceptible to mitochondrial dysfunction due to their complex morphology and high metabolic requirements. Neuronal dysfunction is a central feature of neurodegenerative diseases and is therefore closely related to mitochondrial dysfunction, but its mechanisms have not been fully elucidated (Mamun et al., 2020). Aβ and tau are localized in mitochondria, and Aβ and tau have a direct synergistic toxic effect on synaptic mitochondria (Mamun et al., 2020). At present, neurotoxic drugs targeting Aβ and tau proteins alone have not achieved significant efficacy, so a separate focus on the role of tau or Aβ in AD pathogenesis may not be entirely correct (Wilkins, 2023). It has been shown that in brain tissue samples from *APP* transgenic mice, Aβ preferentially associates with the mitochondria of neurons and the high energy demand site rich in synapses in the hippocampus (Nabi et al., 2022). The Aβ-bound ethanol dehydrogenase (ABAD)-Aβ complex is hypothesized to increase mitochondrial dysfunction and cytotoxicity caused by Aβ through nanomolar affinity directly binding to ABAD. In an Aβ-rich environment, overexpressing ABAD decreases mitochondrial enzyme activity while promoting the generation of free radicals in neurons (Zhang et al., 2021; Wilkins, 2023).

It was demonstrated that Aβ causes the generation of free radicals, increases lipid peroxidation, inhibits mitochondrial inner membrane oxidase activity, decreases ATP production, and ultimately leads to mitochondrial dysfunction, resulting in massive neuronal damage (Akıncıoğlu and Gülçin, 2020). Reddy et al. (2023) used mAPP-expressing HT22 neurons to create a model of AD, and they found that mAPP-expressing HT22 neurons had decreased cell survival, decreased maximal oxygen consumption rate, increased hydrogen peroxide content, decreased ATP production as well as synaptic content, defective mitochondrial function, and decreased synaptic mitochondrial autophagy protein (Dhapola et al., 2022). It has also been shown that alteration of mitochondrial function by COX reduces Aβ production in AD mice; alteration of mitochondrial DNA sequence leads to subsequent changes in mitochondrial function and the size of Aβ plaques (Akıncıoğlu and Gülçin, 2020).

Aging and AD are closely related, and mitochondrial malfunction is one of the signs of aging. Mitochondrial ridge fracture, mitochondrial swelling and a reduction in the number of neurons in the brain tissue of AD patients provide a structural basis for mitochondrial dysfunction within the brains of AD patients (Saxena and Dubey, 2019; Akıncıoğlu and Gülçin, 2020). As age, mitochondria may lose their ability to regulate calcium effectively. It has been shown that Aβ forms calcium permeability pores in the plasma membrane, allowing calcium to accumulate in the cytoplasm, which may lead to excessive calcium uptake by mitochondria, resulting in free radicals and mitochondrial dysfunction (Ashleigh et al., 2023).

Multiple studies have shown a link between tau and mitochondrial dysfunction. Studies have shown that tau overexpression has been shown to induce mitochondrial perinuclear distribution, altered mitochondrial morphology, increased retrograde mitochondrial transport, defective mitochondrial phagocytosis, and decreased complex I and ATP activity (Cheng and Bai, 2018). In *APP/PS1* transgenic mice, researchers found that abnormally phosphorylated tau proteins impair mitochondrial dynamics (Wang et al., 2005; Ittner et al., 2016). Early accumulation of tau protein promotes mitochondrial fusion and later increases mitochondrial cleavage. In AD patients, the pathogenic form of tau protein mostly affects mitochondrial function by limiting ATP generation and reducing antioxidant enzyme activity, which ultimately results in synaptic dysfunction (Ittner et al., 2016). The pathological form of tau protein primarily impairs mitochondrial function by impairing mitochondrial kinetics, transport and biological functions. Other studies have shown that oxidative stress caused by treating neurons with ROS can increase tau phosphorylation; treatment with antioxidants decreased tau protein phosphorylation levels, suggesting that oxidative stress in mitochondria can lead to abnormal tau protein hyperphosphorylation (Cheng and Bai, 2018; Leuzy et al., 2019; Ashleigh et al., 2023).

Mitochondrial autophagy is essential for maintaining mitochondrial and cell homeostasis. In cell senescence and under the action of ROS stress, depolarization damage occurs in mitochondria (Song et al., 2021). In order to maintain the stability of the mitochondrial network and maintain the stability of the intracellular environment, cells use the autophagy mechanism to selectively wrap and degrade damaged or dysfunctional mitochondria in the cell, a process called mitochondrial autophagy (Kerr et al., 2017; Michael Tran, 2021; Song et al., 2021). Therefore, mitochondrial autophagy is an important way for cells to remove damaged or excess mitochondria and maintain the balance of mitochondrial environment (Michael Tran, 2021). Unhealthy mitochondria in the this-clearance pathway are engulfed by autophagosomes, which then fuse with lysosomes to form mitochondrial phagosomes. Over the years, numerous studies have shown a close association between autophagy and AD, with autophagy playing a role in the brain’s homeostasis and clearance of Aβ in physiological environments (Michael Tran, 2021). Autophagy depends on phagocytic degradation by lysosomes, and the number of autophagic vesicles and lysosomes increases in the early stages of the disease, but progressive damage to autophagy occurs due to the failure of timely substrate clearance (Kerr et al., 2017). Activation of mitochondrial autophagy in AD mice lessens the buildup of Aβ in the brain and improves cognitive function in mice. In Guo et al. (2021) demonstrated that OAB-14 can effectively improve lysosomal autophagy dysfunction in AD mice. Endocytosed Aβ was delivered to the lysosome and was hydrolyzed by acid hydrolases within the lysosome. The authors found decreased acid hydrolase activity in the lysosomes of *APP/PS1* transgenic mice, significantly higher levels of the autophagy marker protein p-ULK1, lower levels of Atg5 and beclin1, and accumulation of Aβ, indicating that *APP/PS1* transgenic mice’s autophagy was drastically impaired (Guo et al., 2021). OAB-14 therapy for 3 months resulted in a decline in the number of endosomes in the cells of the *APP/PS1* transgenic mice, an increase in the LC3II/LC3I (autophagy vesicle marker) ratio within the cerebral cortex, and a rise in the p-Ampk/ampk ratio, suggesting that OAB-14A may enhance autophagy flux in mice by regulating MPK/mTOR pathway-mediated endocytosis. In addition, lysosomal hydrolase activity was reversed, and the accumulation of Aβ was reduced (Guo et al., 2021).

Protein and mitDNA accumulation were found in cytoplasm and autophagy vesicles in AD neurons, and oxidative damage of neurons was aggravated (Song et al., 2021). Therefore, it has been speculated that mitochondrial autophagy degradation was impaired in AD patients. Other evidence also suggests that patients with reduced mitochondrial autophagy degradation in the brain have higher levels of total and phosphorylated tau proteins in the brain. More recently, researchers used UrolithinA to activate ubiquitin-dependent mitochondrial autophagy to treat Aβ42 expressing Caenorhabditis elegans and *APP/PS1* mice, ultimately reducing Aβ pathology and improving cognitive function. In addition, UrolithinA reduced mitochondrial damage in microglia, stimulated phagocytic clearance of Aβ plaques and reversed the inflammatory response. UrolithinA treatment also enhanced memory and reduced tau hyperphosphorylation in 3xTgAD mice (Soliman et al., 2021).

## Mitochondrial cascade hypothesis

Since late-onset AD patients have not been found to have any *APP or PS* mutations, it is uncertain what causes sporadic AD to produce excessive amounts of Aβ42 (Mancuso et al., 2007; Fišar, 2022). Later researchers proposed the mitochondrial cascade hypothesis in an attempt to explain how and why sporadic AD occurs. There are three views of the mitochondrial cascade hypothesis: the genes of the parents also determine the biological function of the mitochondrial, and mitochondrial DNA is inherited maternally, so mothers have more influence on their children. AD subjects reported a higher probability of having a mother with psychosis (Swerdlow, 2023). The function of mitochondria changes with age, and it may affect the pathological changes related to AD (Swerdlow and Khan, 2004; Swerdlow et al., 2018). Early illness susceptibility is increased in individuals with low baseline mitochondrial function and a rapid reduction in function (Swerdlow and Khan, 2004; Swerdlow et al., 2010, 2018; Yang Y. et al., 2022; Swerdlow, 2023).

The neurons of AD patients have fewer normal mitochondria, and the mtDNA in the mitochondria is missing (Swerdlow, 2023). Decreased mitochondrial function activates downstream cellular changes observed in late-onset AD. These include Aβ amyloidosis, tau phosphorylation, oxidative stress, synaptic loss, and neurodegeneration. Mitochondrial function influences APP expression, APP processing or Aβ accumulation (Swerdlow et al., 2018; Yang Y. et al., 2022).

## Biomarkers of Alzheimer’s disease

The neurodegeneration of Alzheimer’s disease is usually progressive, with an insidious onset but continuous progression after onset. At present, there is still a lack of effective treatment drugs for AD, and modified therapies targeting the core pathological molecules of AD (Aβ and tau proteins) have shown unsatisfactory therapeutic effects in clinical trials, and the lag in the intervention time may be an important reason for the poor results of these drug trials (Toledo et al., 2011). Therefore, disease-modifying therapies will be more effective if they can be diagnosed and started at an early stage of AD, before symptoms and irreversible pathological changes appear (Huang et al., 2022).

As an objective measurement index, biomarkers can be determined to know the physiological process of the body at present. The ideal biomarker should have high specificity and sensitivity, and the results should be highly accurate. The search for a disease-specific biomarker is clinically important for the identification, early diagnosis and prevention of disease and has become an important focus of current research (Simrén et al., 2021). In recent years, the development of the research technology of CSF blood (For details, see Table 1) and other body fluid markers has promoted the early diagnosis of AD patients by clinicians, and made it possible to identify asymptomatic patients.

**TABLE 1:** Alzheimer’s disease (AD)-related biomarkers.

		CSF	Blood
A	Soluble Aβ oligomers and insoluble amyloid deposits form amyloid plaques in the brain	AD patients have lower levels of Aβ42 in the CSF, and the ratio of Aβ42/Aβ40 was significantly decreased. The accuracy of the combined diagnosis of Aβ42 and Aβ40 was better than that of the single diagnosis	The ratio of Aβ42/Aβ40 in the plasma decreases
T	Abnormal phosphorylated tau protein aggregates and their pathological state	Levels of p-tau181 in the CSF of AD patients are 3.4 times higher than those in the control group.	Levels of p-tau181 protein in the plasma can distinguish AD from other neurodegenerative diseases. P-tau217 also showed good predictability
(N)	Neurodegeneration/ne uronal damage	The levels of NFLs and t-tau in the CSF AD patients were higher than those of normal cognitive function.	The NFL/Aβ42 ratio becomes higher

Currently, levels of Aβ42, t-tau, and p-tau in cerebrospinal fluid are considered the best biomarkers for early diagnosis of AD, and levels of Aβ42 can distinguish between AD and other types of dementia. The researchers found no significant difference in Aβ40 levels between the control group and the AD group, while the ratio of Aβ42/Aβ40 decreased significantly. T-tau reflects nerve damage, and p-tau181 reflects the level of phosphorylated tau protein deposition in the brain. Studies have shown that elevated concentrations of p-tau181 and t-tau in the brain are associated with progressive decline in cognitive function. NFL levels in the cerebrospinal fluid of AD patients are higher than those of normal cognitive function, and as cognitive function declines, NFL concentrations in the cerebrospinal fluid gradually increase. However, invasive sampling methods of the cerebrospinal fluid limit its use in clinical practice. Compared to cerebrospinal fluid blood samples, it is easier to obtain, less invasive, and more reproducible. Aβ42, t-tau, p-tau181 and NFL in blood were closely correlated with Aβ42, t-tau, p-tau181, and NFL in cerebrospinal fluid, and the AUC values of the plasma markers NFL/Aβ42 combined were higher than those of NFL or Aβ42 alone. This suggests that NFL/Aβ42 could serve as a highly accurate biomarker for early diagnosis and detection of disease progression in AD.

The difficulty in the treatment of AD is that its pathogenesis is not completely clear, and it is generally recognized that the imbalance between the generation and clearance of Aβ (Roher et al., 2017). Abnormal levels of Aβ form plaques between neurons in the brain that are neurotoxic, leading to neuronal degeneration. Accurate biomarkers are key to early identification of AD and a prerequisite for effective treatment of the disease. The mainstream biomarkers mainly include Aβ, tau protein and neurofilament light chain (NFL). Aβ is the central biomarker of AD and the main component of amyloid plaques, but it does not directly reflect nerve damage, so Aβ is often used in the early diagnosis of AD (Simrén et al., 2021; Park et al., 2022). More than 70 p-tau proteins have been found in neurodegenerative diseases. Recent studies have shown that p-tau217, p-tau231 and p-tau181 have high specificity in CSF or blood. NFL is a component of the axon skeleton and a biomarker of axonal degeneration (Ritchie et al., 2017; Leuzy et al., 2019). The sensitivity of NFL in CSF or blood is high, and it has been significantly changed before the clinical symptoms of neurodegeneration, and it is significantly increased in various neurodegenerative diseases such as AD, amyotrophic sclerosis, spinal muscular atrophy, multiple sclerosis, and Parkinson’s disease. NFL, as a biomarker, varies at various stages of AD and can be used to monitor its progression (Toledo et al., 2011; McGrowder et al., 2021; Park et al., 2022).

### Cerebrospinal fluid biomarkers

Currently, the primary fluid biomarker for studies on AD is CSF. Because of its direct connection with the central nervous system, CSF can better reflect the situation in the brain, and it has been proven to be an excellent source of information for identifying and quantifying biochemical abnormalities within the brain (Delaby et al., 2023). CSF Aβ42, CSF p-tau and CSF total tau protein (t-tau) are the classic and core clinical biomarkers that have been used in the diagnosis of AD.

The concentration of Aβ in CSF visually reflects the extent of Aβ deposition in the brain parenchyma, so the accuracy of Aβ in CSF for the diagnosis of AD is high. Aβ exists mainly as Aβ40 and Aβ42 subtypes, and under normal conditions, the production and clearance of Aβ40 is in dynamic balance; Aβ42 is neurotoxic and accumulates in AD sufferers’ brains to produce senile plaques, showing good accuracy in detecting AD (Hansson et al., 2018). Pena-Bautista et al. (2023) statistically found decreased concentrations of Aβ42 in the CSF of those with AD, and lower CSF Aβ42 concentrations distinguished AD patients from healthy controls. Different illness conditions had different amounts of Aβ42 in the CSF, and AD could be distinguished from other forms of dementia based on the levels of Aβ42. Aβ40 levels between the control and AD groups did not differ significantly, while the ratio of Aβ42/Aβ40 was considerably lower (Li et al., 2022). In addition, the authors assessed the differential diagnostic ability of biomarkers in the CSF. The authors assessed the differential diagnostic ability of biomarkers in the CSF, and the AUS-ROS value for Aβ42/Aβ40 was 0.980, indicating a statistically significant difference in the Aβ42/Aβ40 ratio between AD and non-AD (Dhapola et al., 2022). In contrast, the AUC-ROS value for Aβ40 was only 0.557, indicating no difference between non-AD and AD participants and no discriminatory ability for AD (Pena-Bautista et al., 2023). The only FDA-approved method for detecting Aβ deposits in the brain is Aβ positron emission tomography (PET), and until PET can be used to detect amyloid deposits, low levels of Aβ42 in the CSF may be an early preclinical indicator of AD (Hansson et al., 2018).

Tau protein aggregation to form NFTs is another characteristic pathological alteration in AD. P-tau and t-tau in the CSF are connected to AD disease, with t-tau reflecting neurological damage and p-tau reflecting the extent of phosphorylated tau protein deposition in the brain. Increased concentrations of phosphorylated tau in the brain have been connected to a progressive reduction in cognitive function, and research has shown that p-tau concentrations in the CSF are 3.4 times larger in AD patients than in healthy controls, while elevated t-tau is not connected to the course of the disease (Toledo et al., 2011; Laske et al., 2017; Müller et al., 2017; Clark et al., 2021; Frank et al., 2022). The assay for p-tau protein in the CSF focused on phosphorylation sites such as Thr181, which showed that the CSF in AD patients had significantly increased (Delaby et al., 2023).

After nerve damage, NFL is released from neurons into the blood and CSF, so NFL reflects neurodegeneration (Laske et al., 2017; Hampel et al., 2018; McGrowder et al., 2021). The development of highly sensitive techniques to detect even low levels of NFL in the blood has made NFL an accessible and reliable biomarker of neurodegenerative disease (Dinoto et al., 2023). NFL is a non-specific marker of neuronal damage and might help with the differential diagnosis of frontotemporal dementia and be useful in the differential diagnosis of frontotemporal dementia, as NFL concentrations in CSF are higher in AD patients than in those with normal cognitive function and increase with cognitive deterioration (Pena-Bautista et al., 2023). In addition, NFL in CSF was significantly correlated with tau protein and Aβ levels, suggesting that NFL could be used as a biomarker for predicting cerebrospinal fluid in AD (Aamodt et al., 2021).

Lleo et al. (2019) collected CSF from 467 Alzheimer’s patients in 2019 for data analysis, and in contrast to previous findings in a study of longitudinal trajectories of CSF, the authors found that levels of three biomarkers, p-tau, t-tau and NFL, increased with age; compared to healthy controls, CSF in AD patients showed a t-tau decreased by 1% compared to healthy controls, while p-tau levels were not altered significantly. Both the concentrations of Aβ42 in CSF and the Aβ42/Aβ40 ratio dropped (Zhang et al., 2022). In addition, the authors found that men had 17% higher NFL levels in their CSF than women did, suggesting that AD may be gender related (Zhang et al., 2022). The older the age in AD, the lower the level of t-tau protein, which is different from the effect of age alone on p-tau and t-tau. As mentioned previously, previous reports have shown that declines in p-tau levels and t-tau protein concentrations in the cerebral fluid of AD patients were not altered in a statistically significant way. In contrast, no significant longitudinal changes in p-tau and t-tau were found in the authors’ assay (Lleo et al., 2019; Pena-Bautista et al., 2023).

Alzheimer’s disease and other types of dementia have similar symptoms and patterns, making it challenging to diagnose AD using CSF Aβ, tau and NFL alone. In 2021, Donovan A. McGrowder, D. A. found that for the diagnosis of AD, the Aβ42/Aβ40 ratio has 82 and 51% specificity and sensitivity, respectively. The specificity and sensitivity of t-tau for differentiating AD from other types of dementia were 90 and 73%, respectively. For the identification of AD, p-tau was more accurate than t-tau protein, the Aβ42/p-tau ratio showed a higher predictive value, and the specificity and sensitivity of the combination of the two were 87 and 95%, respectively, so that the combination of biomarkers may have better diagnostic performance for AD (Hansson et al., 2018; McGrowder et al., 2021). Niklas Mattsson took cerebrospinal fluid from 93 AD patients, 187 MCI patients, and 109 control patients, and investigated the accuracy of t-tau, p-tau, NFL, and neurogranin in their CSF to predict AD (McGrowder et al., 2021). The results showed that the AUC value of T-tau was 80.8%. Ng was 71.4%; The NFL was 77.7%. The AUC of the combined diagnosis of T-tau, Ng and NFL was 85.5%, suggesting that the combined diagnosis could improve the accuracy of CSF prediction (Hansson et al., 2018; McGrowder et al., 2021).

### Plasma biomarkers

Cerebrospinal fluid, which needs to be obtained by lumbar puncture, and blood samples are easier to collect, less intrusive and more repeatable than CSF. There is increasing attention on blood markers, so screening for highly specific markers from blood is important for the early diagnosis of AD (Ritchie et al., 2017). The disease begins with the overproduction and accumulation of antibodies until they finally aggregate and form deposits (Park et al., 2022). At present, numerous studies have indicated that Aβ deposition in the brain results in a reduction in the level of Aβ in the cerebrospinal fluid and in the plasma, making a low concentration of Aβ in the plasma a marker for the early detection of AD (Hampel et al., 2018). A study showed that in patients with brain antibody pathology, the ratio of Aβ42/Aβ40 in blood decreased by 10–20%, while cerebrospinal fluid antibodies decreased by 40–60%. This huge difference in variation can affect the judgment of a positive or negative result in patients (Hampel et al., 2018). In this respect, Aβ42/Aβ40 in the CSF is more diagnostically accurate than Aβ42/Aβ40 in plasma (Hampel et al., 2018). Researchers found that compared with cognitively unimpaired patients, the plasma Aβ42/Aβ40 ratio was reduced in patients with MCI, and the risk of progression to dementia within 2 years in this group was increased by approximately 70% (Doecke et al., 2020). In addition to the differential expression of Aβ42/Aβ40 in the plasma of patients with dementia, Aβ42/Aβ40 is also of certain value in the preclinical diagnosis of AD (Doecke et al., 2020). For example, scientists used single-molecule immunoassay to detect plasma Aβ42 and Aβ40, and found that plasma Aβ42/Aβ40 in subjects with Aβ + was significantly lower than that in subjects with Aβ-, and Aβ42/Aβ40 had higher sensitivity (90%) and specificity (80%) for the identification of these two groups (Doecke et al., 2020).

High concentrations of tau protein are associated with rapid disease progression, and tau protein can undergo cleavage and enter the peripheral blood (Frank et al., 2022; Giacomucci et al., 2022). With the development of detection technology, it is possible to detect tau protein in peripheral blood. While a number of biomarkers have been found to be inaccurate in distinguishing AD from non-AD dementia in clinical applications, researchers have recently demonstrated that blood levels of p-tau181 can distinguish AD from other neurodegenerative illnesses, with results consistent with p-tau protein in CSF (Simrén et al., 2021). This suggests that plasma p-tau181 biomarkers are superior to other biomarkers in distinguishing AD from non-AD dementia diseases (Ritchie et al., 2017). At present, there is a literature suggesting that p-tau181 in plasma is expected to be an important indicator of AD pathology. Plasma p-tau181 analysis was performed in 33 patients with subjective cognitive decline (SCD), 32 patients with MCI, and 14 patients with AD dementia (AD-D) (Giacomucci et al., 2022). Twenty-six patients with SCD, 32 with MCI, and 14 with AD-d also underwent CSF biomarker analyses (Aβ42, Aβ42/40, p-tau, t-tau). And according to the A/T (N) system, when A + is associated with T + (independent of N), they are classified as AD pathological lesions (AP +); When A- (independent of T and N) or A + /T-/N- or A + /T-/N +, they are classified as non-diseased (AP-) (Giacomucci et al., 2022). The results showed that plasma p-tau level in SCD AP + group was higher than that in SCD AP- group (2.85 ± 0.53 vs. 1.73 ± 0.64, *p* < 0.001). The plasma p-tau181 level in MCI AP + group was higher than that in MCI AP- group (4.03 ± 1.07 vs. 2.04 ± 0.87, *p* < 0.001) (Giacomucci et al., 2022). In multiple linear regression analysis, AP status was the only variable affecting plasma p-tau181 (*B* = 1.670 [95% CI 1.097:2.244], *p* < 0.001). Plasma p-tau181 was highly accurate in distinguishing between AP + and AP- patients (AUC = 0.910). Besides, scientists used SIMOA technology to determine the plasma biomarkers of individuals with MCI, AD and cognitive normal (CU), and found that: Compared with CU, the levels of p-tau181, NFL and t-tau in AD dementia group were increased (*p* < 0.001), Aβ42/Aβ40 were decreased (*p* < 0.001), and Aβ42 was not changed. There were significant differences in p-tau181, NFL, t-tau, Aβ42/Aβ40 between AD group and mild cognitive impairment group (*p* < 0.001) (Giacomucci et al., 2022). Only p-tau181 and NFL in MCI group were higher than those in CU group (*p* < 0.001). In addition, the AUC value of plasma p-tau181 was significantly higher than that of other plasma markers. These results indicate that plasma p-tau181 has better diagnostic and prognostic effects than other possible plasma biomarkers for AD (Aβ42/Aβ40, NFL, t-tau), which indicates that p-tau181 is likely to become the most clinically significant plasma biomarker (Giacomucci et al., 2022).

Neurofilament light chain in blood is closely correlated with NFL in CSF, and some studies have confirmed that serum levels of NFL correlate with disease stage and severity of symptoms, suggesting that serum NFL may be an early biomarker for early AD diagnosis (Weston et al., 2017). However, it is difficult to distinguish from other neurodegenerative conditions and may not be an ideal biomarker for identifying AD. Jung Eun Park’s team analyzed plasma biomarker concentrations in healthy controls and AD patients, and the authors discovered that a significant increase in NFL occurred from clinical AD to dementia AD. Plasma NFL did not show significant changes in the AD group, while plasma Aβ42 was noticeably higher in the AD group than in the healthy controls (Aoyagi et al., 2019). The AUC values for the combined plasma marker NFL/Aβ42 were higher than those for NFL or Aβ42 alone, suggesting that NFfL/Aβ42 may serve as a very reliable biomarker for the early diagnosis and monitoring of AD disease development and could be used as an initial screening tool for AD and in subsequent research on the illness’s potential treatment. In addition, NFL/Aβ42 also reflects both neurodegenerative lesions and pathological changes in Aβ42 (Park et al., 2022).

The BBB can prevent the transfer of biomarkers between the ventricles and the blood, resulting in low levels of biomarkers in the blood (Hopperton et al., 2018). Although plasma biomarkers show great potential in the future, their accuracy is lower than that of CSF, and secondly, plasma biomarkers are not suitable for the assessment of specific pathological stages. It is important to identify the pathological stage of AD patients, because different pathological stages have significantly different effects on the future cognitive status of patients. However, plasma biomarkers, because of their low cost and low technical requirements, can be used as a mass screening tool, and can further confirm the pathological status of patients with CSF testing. As the technology develops, plasma biomarkers may be able to compensate for these shortfalls in predictive accuracy, potentially replacing CSF testing.

### Exosomes in Alzheimer’s disease

In recent years, extracellular vesicles have been revealed to be crucial to regular normal cellular physiological functions and in the development of disease. Among them, exosomes are the most studied extracellular vesicles, which are nanosized tiny vesicles secreted by cells into the extracellular matrix under different physiological states. Exosomes can be produced by all brain cells and shuttle in different nerve cells to regulate different neuronal processes, such as neurogenesis, neuroinflammation, and neuronal plasticity (Beatriz et al., 2021). Exosomes can be obtained through urine and saliva, can change early in the lesion and are easily detected, thus making them accessible and non-invasive as a biodiagnostic method.

In AD, it has been suggested that exosomes have a role in the pathophysiology of AD through their participation in the metabolism, dissemination, aggregation and clearance of Aβ and tau protein (Laulagnier et al., 2018). Protein aggregation is a pathogenic characteristic of AD illness, and exosomes can be used to discharge aggregated protein into the extracellular environment, which if impaired in exosomal transport would facilitate the dissemination of folded proteins in the brain. In addition to the regulatory effect of endogenous exosomes on AD, exogenous exosomes also show promising therapeutic potential. Studies on cellular models have demonstrated that exosomes can decrease the expression of Aβ and restore the expression of genes related to neuronal memory and synaptic plasticity. In addition, researchers have found that injection of exosomes from the cerebrospinal fluid of AD mice into WT mice resulted in an increase in tau protein in the body fluids of WT mice (Beatriz et al., 2021). In human neuron cultures, most of the secreted tau protein was free-floating, and small amounts were detected in extracellular vesicles, suggesting that there may be multiple mechanisms leading to tau release in cell cultures (Tracy et al., 2022). Exosomes not only spread toxic proteins but also promote their pathological aggregation and then spread them to lesion-free areas where neurons engulf the encapsulated lesion proteins, accelerating neuronal degeneration and thus creating a vicious cycle.

Exosomes play a significant role in physiological processes such as the inflammatory response, immune response, biological development and the exchange of information between cells (Pakravan et al., 2017). In a 5xFAD-AD mouse model, mesenchymal stem cell (MSC)-derived EVs were shown to save cognitive decline (Pakravan et al., 2017). MSC-EVs treatment reduced neuronal loss, favored neuronal function, increased neurogenesis, improved synaptic morphology, etc., and restored brain levels of various synaptic proteins, including synaptophysin, to those of WT mice. These findings highlight the powerful efficacy of stem cell-derived EVs in alleviating symptoms of AD (Pakravan et al., 2017).

The BBB is a protective barrier that can ward off the threat of harmful substances and restrict the transport of large molecular proteins. Exosomes, as lipid vesicles that are hydrophobic and low in water solubility, can cross the BBB and act as biological transport carriers, thus allowing them to be used as drug carriers for therapeutic purposes (Soliman et al., 2021). Since exosomes can transport a variety of nucleic acid components, exosome-based drug delivery systems have promising applications. Yang Y. et al. (2022) found that exosomal administration of miR-124a improved glutamate uptake and prevented neuronal apoptosis in mice by increasing the expression of excitatory amino acid transporter protein 2 on the surface of astrocytes (Morel et al., 2013). Although EVs can cross the BBB, studies have shown that peripherally administered EVs accumulate primarily in peripheral organs (e.g., liver, lung, kidney, spleen), while brain delivery is limited.

### Gut microbiota and AD

It is well known that the gut microbiota plays an important role in pathogen defense, immunity, and nutrient acquisition. Multiple pieces of evidence suggest that the dynamic interactions among the gut microbiota, the intestinal epithelial barrier, and the gut-neuroimmune network may be the starting point of neurodegenerative processes (Wang et al., 2019). Changes in the composition of the gut microbiota lead to increased intestinal barrier, and promotion of neuroinflammation, which are potential mechanisms underlying neurodegenerative diseases (Braniste et al., 2014; Anderson, 2022). The gut-brain axis significantly contributes to regulating bidirectional communication between the gut microbiota and the brain (Braniste et al., 2014; Erny et al., 2015; Petra et al., 2015). High levels of stress in the central nervous system may disrupt gut physiological functions and result in an imbalance of the gut microbiota (Petra et al., 2015). Moreover, the gut microbiota can influence brain function and bioactivity through various pathways. The changes in gut microbial composition lead to alterations in microbial metabolites, which modulate the maturation, differentiation, and activation of astrocytes and microglia, ultimately mediating the activation of the central nervous immune system.

D’Argenio et al. (2022) investigated the changes in bacterial and fungal composition in the intestines of wild-type (WT) mice and AD (3xTg-AD mice, one of the most widely used AD models). Compared to WT mice, AD mice showed reduced relative abundance of the phyla Firmicutes, Lachnospiraceae-unclassified, Ruminococcaceae-unclassified, and Turicibacter. Among them, the Coprococcus genus is an anti-inflammatory bacterium, belonging to the Firmicutes phylum, and previous studies have also found its reduction in brain-related diseases. The relative abundance of the phyla Bacteroidetes, Lactobacillus, and Parasutterella increased in AD mice. Bacteroidetes is a gram-negative bacteria and the lipopolysaccharide in its outer membrane has pro-inflammatory effects and promotes amyloid protein deposition and tau protein-related pathology. In addition, the authors found a decrease in fungal richness in the feces of AD mice, which may be related to metabolic changes in AD (D’Argenio et al., 2022). Liang et al. (2023) transplanted Paenalcaligenes hominis isolated from the feces of elderly humans and mice into young mice and found a decline in cognitive function in the mice.

Changes in the gut microbiota can lead to ecological imbalances, triggering intestinal dysfunction, which can lead to neuroinflammation. Cognitive improvement is associated with a reduction in inflammatory response, and studies are showing that probiotic treatment can improve cognitive performance. Pathologically, Aβ deposition is the main pathological feature of AD. Extensive data suggest that alterations in the microbiota in AD model mice promote the deposition of Aβ protein in the brain (Zhu et al., 2020). It has been found that in AD transgenic mice, the intestinal vascular Aβ deposition, intestinal microbiota disorder and AD remain unclear (Liu et al., 2020).

## Future and outlook

Due to the complex etiology and highly heterogeneous pathological changes of AD, it is difficult to adequately reflect the pathological changes and make an accurate diagnosis of the disease using a single biomarker; therefore, a combination of multiple markers is often needed for the comprehensive diagnosis of AD sufferers. In recent years, new technologies such as proteomics have helped researchers find and screen out many novel AD candidate markers, greatly expanding the scope of AD markers. More humoral biomarkers will be widely validated and eventually incorporated into the biological diagnostic framework of AD, and humoral biomarkers will play an important role in the pathogenesis, early diagnosis, treatment guidance and efficacy evaluation of AD.

## Discussion

It is important to investigate the etiology and pathogenesis of AD to explore therapeutic approaches to prevent and treat AD, which is a major problem that needs to be addressed. The pathological features of AD are nerve spots and neuronal tangles caused by abnormal deposition of Aβ and tau proteins. Therefore, the researchers attributed the cause of AD to Aβ misfolding and tau protein hyperphosphorylation. The amyloid hypothesis is the mainstream theory to explain the pathogenesis of AD, but many studies have questioned this hypothesis. First, there is a time lag of several decades between the onset of Aβ deposition in the brain and the onset of AD in patients, and second, although AD mice have a large amount of Aβ deposition in the brain at an advanced age, neuronal death is not obvious or even very mild. These studies suggest that amyloid deposition may be a necessary and insufficient condition for the development of AD. In addition, the clinical drugs developed for Aβ have failed in clinical trials, which has also prompted scientists to find new targets for AD treatment.

Abnormal deposition of Aβ and abnormal phosphorylation of tau protein are currently recognized as the main molecular mechanisms of AD, and there is a certain correlation between Aβ and tau proteins, which jointly mediate the course of AD. In addition to the above three hypotheses, there are other hypotheses such as prion-like mechanism, inflammatory response, mitochondrial cascade hypothesis, calcium imbalance hypothesis, brain-gut axis hypothesis, etc., which are difficult to independently explain the cause of AD. Mitochondria produce the necessary ATP to maintain the survival of neurons and their optimal function, and mitochondrial dysfunction is closely associated with aging and neurodegenerative diseases. In AD, the dysfunction of neuronal mitochondria can lead to typical Aβ deposition and hyperphosphorylated tau pathology. In turn, Aβ deposition and tau pathology further promote this defect in mitochondria. Energy deficiency and Aβ42 oligomers caused by mitochondrial dysfunction trigger intracellular Ca^2+^ imbalance and AMPK activation, ultimately leading to synaptic toxicity and memory loss. Aβ42 and p-tau induce axon transport dysfunction, leading to synaptic starvation, ATP depletion, and ultimately neurodegeneration. Therefore, there is a complex association between mitochondrial damage and the two pathologies of Aβ and tau, suggesting that targeting defective mitochondria may provide important evidence for the treatment of AD. The incidence of AD increases with age; Meanwhile, dysfunctional mitochondria and damaged mitochondrial autophagy also accumulate with age. However, the specific role of mitochondrial autophagy in the progression of AD remains unclear.

In recent years, prion protein and the transmission of neurodegenerative diseases have been a focus of research on the pathogenesis of these diseases. At present, neurodegeneration is believed to be caused by a variety of protein conformation changes, and the specific spatial conformation is the structural basis for proteins to perform their biological functions. Although the phenomenon of “self-transmission” of misfolded proteins does exist in AD, Parkinson’s disease and frontotemporal dementia, it is not yet fully proven that these neurodegenerative diseases are prion proteins. In conclusion, new insights into the theory of protein misfolding and protein “self-propagation” not only play an important role in the pathogenesis of neurodegenerative diseases but also may provide new targets for the treatment of such diseases.

Gut microbes have been implicated in the pathogenesis of neurodegenerative diseases. Metabolites produced by gut microbes act as chemical messengers to regulate the interactions between microbes and hosts. Changes in the structural composition and diversity of gut flora will affect the ecological balance of gut flora, and their homeostasis disturbance will induce structural changes and functional network connectivity degradation in the brain. Therefore, gut flora may be an important target for the pathological process of AD. Therefore it is necessary to develop intervention strategies targeting intestinal flora in order to delay or improve the course of AD and provide new intervention ideas for the rehabilitation of nervous system diseases. However, the dysregulation of intestinal flora is rarely reported in AD, and the causal relationship between them and cognitive decline in AD remains to be further verified.

The current non-biomarker diagnostic modalities include MMSE, MRI, and ^18^FDG-PET. These three methods do not work until patients have progressed almost to the middle to late stages of AD, requiring high data analysis skills from researchers, and the results of the experiments are highly volatile depending on the brand of instrument, parameter settings and data analysis, and it is not possible to distinguish AD from other causes of dementia using only non-biomarker assays. In contrast, biomarkers have received much interest in recent years due to their early diagnostic stage, lower cost and higher sensitivity. Most of the current research on AD biomarkers has focused on CSF. CSF has obvious advantages due to the direct exchange of substances with the brain, but few people are willing to use this invasive method for diagnosis and prevention before the onset of obvious dementia symptoms. Studying changes in blood biomarkers, on the other hand, can be a good solution to existing problems. One of the most problematic aspects of studying blood biomarkers is how to accurately and efficiently analyze the target protein without the influence of other miscellaneous proteins. Exosomes are small vesicles secreted by cells that contain a high protein content and rely on their own phospholipid bilayer membrane, which gives them a great advantage when crossing the BBB.

Although AD biomarker research and clinical application have made some progress, there are still many problems and challenges, such as the lack of international uniform measurement standards; the combination of multiple markers can improve the accuracy of the diagnosis of the risk and course of AD, but more research is required to determine the combination of markers that produces the best benefit; and the collection has not yet been standardized with a uniform operational procedure. Therefore, it is important to pay attention to the abovementioned issues, to standardize the collection of biomarkers as much as possible and to establish the same measurement standards so that the final results can be more reliable and referrible and thus better used for the diagnosis and forecast of AD progression.

## Author contributions

HW: Writing – original draft. MS: Writing – review and editing, Conceptualization. WL: Writing – review and editing, Formal analysis. XL: Writing – review and editing, Methodology. MZ: Writing – review and editing, Resources. HQ: Writing – review and editing.
